# The Putative E3 Ubiquitin Ligase TEX1 Is Required for Nuclear Biology and Developmental Progression of *Plasmodium berghei* in the Liver

**DOI:** 10.3390/cells15020155

**Published:** 2026-01-15

**Authors:** Melanie Schmid, Raphael Golomingi, Blandine Franke-Fayard, Reto Caldelari, Ruth Rehmann, Magali Roques, Volker T. Heussler

**Affiliations:** 1 Institute of Cell Biology, University of Bern, Baltzerstrasse 4, 3012 Bern, Switzerlandmagali.roques@unibe.ch (M.R.); 2 Graduate School for Cellular and Biomedical Sciences, University of Bern, 3012 Bern, Switzerland; 3 Leiden Malaria Research Group, Parasitology, Center of Infectious Diseases, Leiden University Medical Center (LUMC), 2333 ZA Leiden, The Netherlands

**Keywords:** malaria, *Plasmodium*, mouse model, parasite liver stage, nuclear division

## Abstract

**Highlights:**

**What are the main findings?**

- TEX1 knockout parasites display severe defects in nuclear division, organelle segregation, and developmental progression during mosquito and liver stages.
- C-terminal HA tagging preserves TEX1 function and reveals a strong, discrete subnuclear localization pattern during liver stage development.

**What are the implications of the main findings?**

- TEX1 is a critical regulator of parasite nuclear biology and liver stage schizogony, and its precise localization supports a role in chromatin- or division-associated processes.
- Disruption of TEX1 effectively blocks the transition from liver to blood stage, highlighting TEX1 as a promising target for liver stage and transmission-blocking interventions.

**Abstract:**

Malaria remains a major global health burden, and the emergence of resistance to blood stage antimalarials underscores the need for new interventions targeting earlier stages of the parasite’s life cycle. The pre-erythrocytic liver stage represents a critical bottleneck and an attractive target for chemotherapeutic and prophylactic interventions. In this study, we functionally characterized the putative E3 ubiquitin ligase Trophozoite Exported Protein 1 (TEX1; PBANKA_0102200) in *Plasmodium berghei* using gene knockout, tagging, and imaging approaches across the mosquito and liver stages. TEX1 knockout parasites (*Pb*TEX1-KO) showed impaired development during mosquito-stage transitions, with significant reductions in ookinete formation, oocyst numbers, and sporozoites reaching the salivary glands. In hepatic stages, TEX1-KO parasites displayed reduced growth, abnormal nuclear division, and impaired liver stage maturation, ultimately leading to a dramatic decline in detached cell formation and blood stage infectivity. Endogenous C-terminal tagging of TEX1 with GFP and 3×HA revealed a discrete subnuclear localization pattern, indicating a critical role in DNA synthesis and/or mitotic regulation. Our findings reveal that TEX1 is required for nuclear replication and division and successful development in both the mosquito and liver stages of *Plasmodium*. Given its pivotal role and nuclear localization during hepatic schizogony, TEX1 represents a promising target for the development of liver stage antimalarial interventions.

## 1. Introduction

Malaria is a life-threatening disease caused by protozoan parasites of the genus *Plasmodium*. Despite advances in prevention and treatment, malaria continues to exert a heavy toll on global health, with approximately 600,000 deaths annually, primarily among children under five years of age [1]. Human infection occurs when an infected *Anopheles* mosquito injects typically fewer than 100 sporozoites into the skin during a blood meal [2,3,4,5,6,7]. Some of these sporozoites enter the bloodstream and migrate to the liver where they traverse Kupffer cells and several hepatocytes before establishing an infection in a single hepatocyte [8,9,10,11,12]. Once inside a host cell, the sporozoite develops into a liver stage schizont, undergoing multiple rounds of nuclear replication and partial mitosis, to ultimately produce thousands of daughter merozoites [13,14,15].

This extensive nuclear division is a hallmark of the liver stage and represents a major amplification step in the parasite life cycle. Efficient nuclear replication is crucial for producing a sufficient number of merozoites to establish a successful blood stage infection. Errors in this process can compromise parasite viability and transmission. Liver stage merozoites are eventually released in merosomes into the bloodstream, where they initiate the erythrocytic cycle, a stage responsible for malaria symptoms and further transmission [15,16,17,18,19].

The liver stage is clinically silent but essential for parasite establishment and disease progression, making it an attractive target for antimalarial drug and vaccine development. Recent efforts to better understand the molecular machinery regulating liver stage development have highlighted the importance of processes such as organelle segregation, chromatin remodeling [20], and nuclear division [21]. However, the molecular factors coordinating these events remain incompletely understood.

In a large-scale gene knockout screen [22] in *Plasmodium berghei*, the E3 ubiquitin ligase Trophozoite Exported Protein 1 (TEX1; PBANKA_0102200) emerged as a gene of interest due to its impact on parasite fitness across multiple life cycle stages. Interestingly, TEX1 deletion caused no major growth defect during asexual blood-stage development [22] (blood stage screen in *P. berghei*) but a pronounced loss of fitness during mosquito and liver stages. TEX1 is a putative E3 ubiquitin ligase predicted to contain a RING-like zinc-binding domain (Supplementary Figure S1A,B). E3 ligases act by recruiting ubiquitin-loaded E2 enzymes to their substrates, promoting ubiquitin transfer and, in many cases, targeting proteins for proteasomal degradation [23]. Ubiquitination in *Plasmodium* has been implicated in regulating protein stability [24], trafficking [25], and gene expression [26,27], particularly during stages involving major cellular reprogramming.

Orthologs of TEX1 have been identified in *P. falciparum* and other species [28,29,30], and its transcript level peak during blood schizogony and liver stage development, suggesting a role in parasite development at those stages [31]. Yet, the specific function of TEX1 and its contribution to parasite development during hepatic schizogony has not been previously explored.

Here, we investigate the role of TEX1 in *P. berghei*, focusing on its function during mosquito and liver stage development. Using gene knockout and protein tagging approaches, we demonstrate that TEX1 is required for proper DNA replication and segregation and progression through hepatic schizogony. Our findings provide new insight into the regulation of parasite replication and identify TEX1 as a potential target for liver stage antimalarial intervention.

## 2. Material and Methods

### 2.1. Experimental Animals

Animal experiments were performed with strict accordance to the guidelines of the Swiss Tierschutzgesetz (TSchG; Animal Rights Laws) and were approved by cantonal authorities in Bern (experimental license BE118/2022).

All mice were housed in individually ventilated cages (Techniplast S.p.A. Buguggiate, Italy) equipped with autoclaved aspen bedding, mouse house and paper towels for nesting. Mice were kept in a 12 h light/dark cycle at 20–22 °C and 55 ± 10% relative humidity (RH). The animals were fed ad libitum with standard autoclaved dry rodent chow and tap water. Regular tests, including sentinel screening, confirmed their hygienic status.

6–12-week-old BALB/c mice (Janvier Laboratories, Le Genest-Saint-Isle, France or in house breeding) were injected intraperitoneally with blood stabilates for the parasite maintenance. At a parasitemia of 2% to 6% (measured by flow cytometry), mice were euthanized in a CO_2_ chamber, parasites were isolated by cardiac puncture and blood was passaged into new mice to for mosquito blood feeding. At around 0.5% gametocytemia mice were anaesthetized (ketamine:xylazine) and made available to approximately 100–150 mosquitoes for a blood meal.

For gametocyte fertilization analysis, 6–12-week-old BALB/c mice (Janvier Laboratories, Le Genest-Saint-Isle, France or in house breeding) were intraperitoneally injected with 200 µL Phenylhydrazine (PHZ, Sigma-Aldrich, 114715) to induce hemolytic anemia and stimulate erythropoiesis. 3 days after PHZ treatment, *P. berghei* parasites were passaged into the PHZ-treated mice to obtain high parasitemia levels.

For in vivo liver stage experiments, 5000 salivary gland sporozoites were injected intravenously in female 8–10-week-old C57BL/6JRj mice (Janvier laboratories, France). Female mice were used to minimize interindividual variability and stress caused by single housing. Mouse blood samples were taken daily from day 2 to day 19 to evaluate parasitemia by flow cytometry. To prevent the occurrence of cerebral malaria mice were euthanized when reaching a 2% parasitemia. All infected mice were checked daily according to approved score sheet.

All animals were euthanized by CO_2_ inhalation followed by exsanguination, in accordance with the ethical guidelines.

### 2.2. Mosquito Breeding

*Anopheles stephensi* mosquitoes were kept in an insectary under a 12 h light-dark cycle at 28 °C and 80% humidity. They were fed daily with 8% fructose and 4-Aminobenzoic acid (pABA) (0.2 g/L, A9878, Sigma-Aldrich, Buchs, Switzerland) solution presented on cotton pads. Infected mosquitoes, after blood meal on infected mice, were kept at 20.5 °C and 80% RH.

### 2.3. Generation of Transgenic Parasites

To exclude the possibility of clone-specific effects, TEX1 knockouts were generated in two genetically similar parasite lines, allowing us to verify that the observed phenotype is consistent and reproducible.

#### 2.3.1. TEX1-KO Parasites

The *P. berghei* ANKA reporter line (*Pb*1868) was transfected using a standard transfection method [32] with *Pb*GEM-266212-KO vector (PlasmoGEM [33,34]) to replace the PBANKA_0102200 gene with a KO cassette [35] containing a gene-specific barcode and a selection marker cassette (3xHA-hdhfr-yFCU) (see Figure S2A). Pyrimethamine, administrated via drinking water, was used to positively select for transgenic parasites. Limiting dilution [36] of the transgenic TEX1-KO parasites was performed in mice, to obtain a clonal KO parasite line. Successful gene replacement was confirmed by integration PCR with primer pairs specific for wildtype (REV: 5′-AAAGGGCCCATCTAAAAACATTTTTTGCAAATCC-3′ + GT: 5′-CACAGCCAAATAAGGTTTAAC-3′) or KO (GT: 5′-CACAGCCAAATAAGGTTTAAC-3′ + GW2: 5′-CTTTGGTGACAGATACTAC-3′).

#### 2.3.2. TEX1-KO GOMO Parasites

The wildtype *P. berghei* ANKA parasite line (*Pb*WT) [37] was transfected [32] with barcoded *Pb*GEM-645656-KO vector from PlasmoGEM [33,34] was used to replace the *PB*ANKA_0102200 gene with a triple marker cassette (GFP/hDHFR-yFCU/mCherry) (see Figure S2B). Pyrimethamine, administrated via drinking water, was used to positively select for transgenic parasites. Limiting dilution [36] of the transgenic TEX1-KO-GOMO parasites was performed in mice, to obtain a clonal KO parasite line. Successful gene replacement was confirmed by integration PCR with primers specific for wildtype (REV: 5′-AAAGGGCCCATCTAAAAACATTTTTTGCAAATCC-3′ + GT: 5′-CACAGCCAAATAAGGTTTAAC-3′) and KO (GT: 5′-CACAGCCAAATAAGGTTTAAC-3′ + GW2.2: 5′-GAAAACTGAGGGAATATACACTGTAGATCC-3′).

#### 2.3.3. TEX1-3xHA Parasites

The C-terminal part of the TEX1 gene was amplified by PCR and cloned into a parent plasmid (pDHR) upstream of a 3x HA tag to generate the vector pDHR-5′HR TEX1-3xHA-mCherry-3′HR TEX1. The plasmid was linearized by SnaBI and transfected [32] into wildtype *P. berghei* ANKA parasite line (*Pb*WT) to allow integration by double homologous recombination into the endogenous locus. Transgenic parasites were positively selected with pyrimethamine. Successful integration of the construct was verified by flow cytometry (mCherry) and integration PCR (FOR: 5′-AAAGTGTTTTAAGAAATATCAATGTAG-3′, REV2.1: 5′-ATCTAAAAACATTTTTTGCAAATCC-3′ and REV2.2: 5′-ACCAACCATGGTACCCCCTATGTTTTATAAAAT-3′).

#### 2.3.4. TEX1-EGFP Parasites

The C-terminal part of the TEX1 gene was amplified by PCR and cloned into a parent plasmid (pOB277) upstream of an EGFP tag to generate the vector TEX1-EGFP. The plasmid was linearized by EcoRV and transfected [32] into *Pb*1868 parasite line to allow integration by single homologous recombination into the endogenous locus. Transgenic parasites were positively selected with pyrimethamine. Successful integration of the construct was verified by flow cytometry (EGFP) and integration PCR (FOR: 5′-AAAGTGTTTTAAGAAATATCAATGTAG-3′ and REV3.1: 5′-AAAGGGCCCATCTAAAAACATTTTTTGCAAATCC-3′).

### 2.4. Analysis of Gametocyte to Ookinete Progression

An amount of 10 μL of fresh blood from a PHZ-treated mouse was transferred to 50 μL of ookinete medium (RPMI1640, 25 mM Hepes, L-glutamine, 2 g sodium bicarbonate, 10 mL Pen/Strep solution, 20 mg xanthurenic acid, with 20% FCS at pH 7.8–8). After incubation for 12–15 min at RT, exflagellation centers in 10 fields of view (FOV) were counted by microscopy (Nikon Eclipse E600, 63x objective).

To evaluate ookinetes formation blood was incubated at 20 °C for 24 h, stained with Hoechst33342 (B2261, 20 μM in 1x PBS, Sigma-Aldrich, Buchs, Switzerland) and ookinetes were counted in 10 FOVs by microscopy (Leica DM 5500, 63x objective, Leica Microsystems, Wetzlar, Germany).

### 2.5. Live Imaging and Quantification of Midgut Oocysts

Midguts from infected mosquitoes were carefully dissected on day 6/7 and 14 post-feed, imaged by microscopy (Leica DM6000, 5x objective, Leica Microsystems, Wetzlar, Germany) and evaluated using Fiji ImageJ software version 2.16.0. For this, the TX2 channel (mCherry) was processed to generate a binary image. Overlapping oocysts signals were separated with the watershed function and the individual particles were analyzed. Graphs and statistical analysis were performed with GraphPad Prism 10.2.3. To visualize oocysts at days 7 and 14 were stained with Hoechst33342 (1:500) and visualized with the Leica TCS SP8 confocal microscope using the 63x objective. Image processing was performed using Fiji ImageJ.

### 2.6. Salivary Gland Sporozoite Infection of Wildtype HeLa Cells In Vitro or Mice In Vivo

Wildtype HeLa (European Cell Culture Collection) cells were cultivated in Minimum Essential Medium with Earle’s salts (MEM EBS; 1-31F01-I, Bioconcept, Allschwil, Switzerland) supplemented with 10% FCS, 100 U penicillin, 100 μg/mL streptomycin, and 2 mM L-glutamine (all from Bioconcept). Cells were grown at 37˚C with 5% CO_2_ and split using Accutase (Innovative Cell Technologies, San Diego, CA, USA). 40,000 pre-seeded HeLa cells were infected with approximately 10,000 to 20,000 *P. berghei* sporozoites and maintained in supplemented medium containing 2.5 µg/mL amphotericin B (E437, Bioconcept, Allschwil, Switzerland) to prevent fungal contamination. Infections were performed in three independent experiments, each conducted in triplicates. The medium was renewed daily.

Salivary glands of infected mosquitos were dissected in Iscove’s Modified Dulbecco’s Media (IMDM, Bioconcept, Allschwil, Switzerland) and homogenized to release the sporozoites. They were quantified with Neubauer counting chamber and either added to cultured cells to evaluate parasite development in vitro or injected intravenously into C57BL/6 mice.

### 2.7. Automated Microscopy

To measure parasite survival over time and parasite size infected HeLa cells were analyzed by automated microscopy (GE Healthcare InCell Analyzer 2000, 10x objective, GE HealthCare, Düsseldorf, Germany) at different time points (6, 24, 48, and 56 h post-infection (hpi)) Image analysis was carried out using the InCell Developer tool 1.10.0 software by defining parameters such as kernel size, sensitivity, and a range for parasite size to detect individual parasites. The mCherry signal was conducted in “object” mode, particles smaller than 10 μm^2^ or larger than 600 μm^2^ were excluded. Absolute parasite numbers at defined time points were used to calculate the percentage of survival over time. Graphs and statistical evaluations were done with GraphPad Prism version 10.2.3.

### 2.8. Detached Cell Formation Analysis

To analyze the completion of liver stage development confluent HeLa cells (seeded at 40,000 cells per 96-well, on the day before infection) were infected in triplicates with 20,000 salivary gland sporozoites for 2 h. The cells of each well were released with Accutase. Cells from each well were seeded into 8 wells on two 96-well plates (i.e., 4 wells per plate). The cells from one plate were fixed with 4% PFA in 1 x PBS for 10 min at 48 hpi. Parasite numbers were determined by microscopy (GE Healthcare InCell Analyzer 2000 10x objective). At 65 hpi, detached cells in the second plate were transferred to a fresh plate by gentle pipetting and counted by fluorescence microscopy (Olympus CKX41, Ryf AG, Grenchen, Switzerland). The experiment was conducted in triplicates and repeated independently twice.

The number of detached cells per infection triplicate was divided by the number of parasites counted at 48 hpi to obtain the detached cell formation rate in percent [22].

### 2.9. Indirect Immunofluorescence Assays (IFA)

#### 2.9.1. Sporozoite Stage

Dissected sporozoites were seeded on coverslips in a 24-well plate, centrifuged at 1000 rpm for 1 min, and incubated with complete MEM for 2 h at 37 °C. The sample was subsequently fixed with 4% PFA/PBS for 10 min, washed with PBS, permeabilized with 0.1% Triton X-100 for 5 min, washed with PBS, blocked with 3% BSA/PBS for 1 h, incubated for 60 min hours with rabbit anti-*Pb*CSP antibodies (1:500 in 3% BSA/PBS, Eurogentec, Seraing, Belgium) and subsequently with goat-anti-Rabbit-Alexa488 antibody (1:2000 in 3% BSA/PBS, Invitrogen by Life Technologies Europe BV, Reinach, Switzerland). Nuclei were stained with DAPI (1 μg/mL) for 10 min. The coverslips were mounted on a microscope slide in 5 μL fluorescent mounting medium (S3023, Agilent Technologies, Santa Clara, CA, USA) and sporozoites were imaged by confocal microscopy (Leica TCS SP8 laser-scanning confocal, 63x objective, Leica Microsystems, Wetzlar, Germany).

#### 2.9.2. Exo-Erythrocytic Stages

Infected Hela cells grown on coverslips were fixed with 4% PFA/PBS for 15 min at RT, permeabilized with 1 mL ice-cold 100% ice-cold methanol for 30 min at −20 °C. 10% FCS/PBS was used to block unspecific antibody binding. Cells were then incubated with the primary antibodies diluted in 10% FCS/PBS for 1–3 h at RT. Primary antibodies used were rabbit anti-UIS4 (1:1000, Proteogenix, Schiltigheim, France), rat α-*Pb*ACP (1:50, custom-made), mouse α-HA (F-7, 1:1000, Santa Cruz Biotechnology, Dallas, TX, USA) and rabbit anti-*Tg*Hsp70 (1:250 kindly gifted by Dominique Soldati). Cells were then washed with PBS and incubated for 1 h with secondary antibodies diluted in 10% FCS/PBS plus DAPI as nuclear stain. The secondary antibodies used were goat anti-rabbit Alexa488 (A-11008; 1:2000, Invitrogen by Life Technologies Europe BV, Reinach, Switzerland), goat anti-rat Alexa647 (Invitrogen A-21247; 1:2000), goat anti-mouse Alexa488 (Invitrogen A-11001, 1:2000) and goat anti-rabbit Cy5 (1:2000, Dianova by Biozol, Hamburg, Germany). Coverslips were mounted with 5 μL fluorescent mounting medium and examined by confocal microscopy (Leica TCS SP8 laser-scanning confocal, 63x objective, Leica Microsystems, Wetzlar, Germany).

## 3. Results

### 3.1. TEX1 Is Required for Sexual Development and Oocyst Maturation in the Mosquito Stage

To investigate the function of TEX1 during the mosquito stage of *Plasmodium berghei*, we generated a TEX1 knockout (*Pb*TEX1-KO) parasite line using the barcoded *Pb*GEM-266212-KO vector [33,34], which was transfected into schizonts of the fluorescent reporter line *Pb*1868 [38]. This line expresses mCherry under the control of the *hsp70* promoter and luciferase under the *eef1α* promoter. The virulent wildtype development of *Pb*1868 parasites made them a suitable control throughout the study.

Using double homologous recombination, the endogenous PBANKA_0102200 locus was replaced with a selection cassette containing a gene-specific barcode and a 3xHA-hDHFR-yFCU marker (Supplementary Figure S2A). A clonal TEX1-KO line was obtained by limiting dilution and used for subsequent phenotypic analysis.

**Figure 1:**
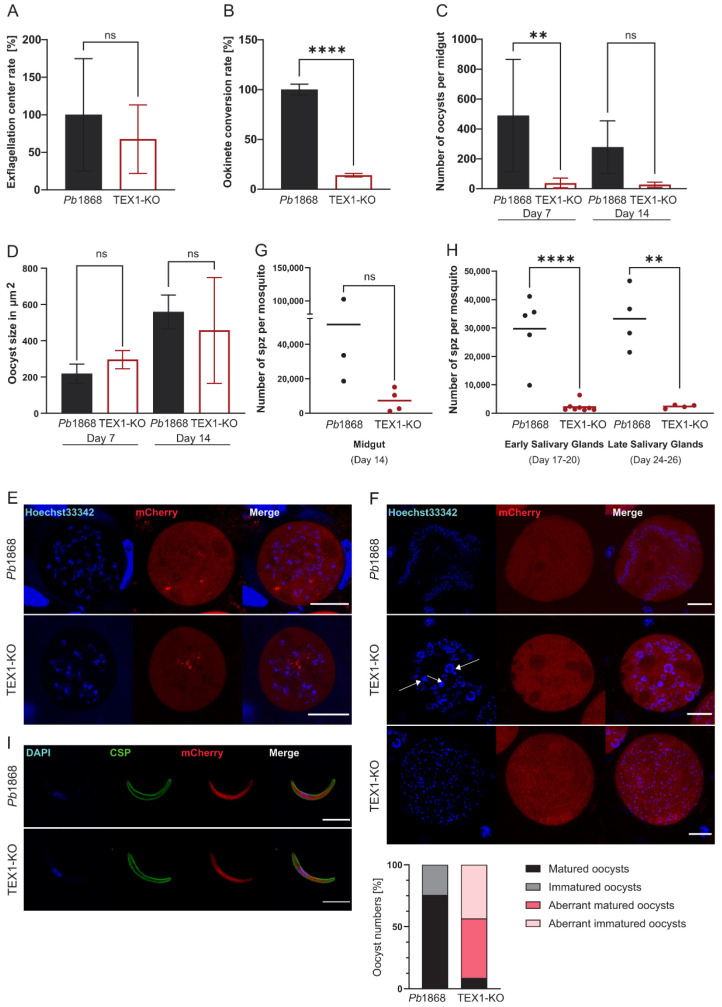
TEX1 is required for sexual development and sporogony in *Plasmodium berghei.* (**A**) Quantification of exflagellation centers formed by male gametocytes of *Pb*1868 and TEX1-KO parasites, 12–15 min after activation in ookinete medium in vitro. No significant difference was observed (Students *t*-Test; ns = not significant; n = counts in 10 fields of view (FOV)). (**B**) Quantification of ookinetes formed in vitro by *Pb*1868 and TEX1-KO parasites 24 h post-activation in ookinete medium (same cultures analyzed as in A, Students *t*-Test; **** = *p* < 0.0001; n = counts in 20 FOV). (**C**) Quantification of midgut oocysts from *Pb*1868 and TEX1-KO-infected mosquitoes on days 7 and 14 post blood meal (one-way ANOVA; ns = not significant; ** = *p* < 0.01; n = 10 midguts per group). (**D**) Measurement of oocyst size (area, μm^2^) at day 7 and day 14 post infection. No significant difference in oocyst size was detected between *Pb*1868 and TEX1-KO parasites (one-way ANOVA; ns = not significant; n = 10 midguts (data from panel (**C**)). (**E**,**F**) Representative live-cell images of oocysts at days 7 (**E**) and 14 post-mosquito feed ((**F**) with quantification of oocyst phenotypes). Hoechst33342 (DNA, blue), cytosolic mCherry (red) merged images shown. (**G**) Quantification of midgut sporozoites at day 14 post-feed. TEX1-KO parasites show reduced sporozoite numbers (Students *t*-Test; ns = not significant; n = 5–10 mosquitoes per data point). (**H**) Quantification of salivary gland sporozoites (spz) at days 17–20 (early) and at days 24–26 (late) (Students *t*-Test; ** = *p* < 0.01; **** = *p* < 0.0001; n = 10–30 mosquitoes per data point). (**I**) Representative IFA images of salivary gland sporozoites. CSP (green), DAPI (blue), mCherry (red). TEX1-KO sporozoites display normal morphology and surface CSP localization. Scale bars = 5 μm. Error bars in (**A**–**D**) represent standard deviation (SD).

We first assessed whether TEX1 was required for gametocyte activation and fertilization. The number of male gametocytes undergoing exflagellation in vitro did not differ significantly between TEX1-KO and control parasites (Figure 1A). However, subsequent development into ookinetes was severely impaired in the knockout line: only ~34% of TEX1-KO parasites developed into reproductive forms compared to controls (Figure 1B).

To determine whether TEX1-KO ookinetes can develop into oocysts, mosquitoes were fed on infected mice, and midgut oocysts quantified on days 7 and 14 post-feed. TEX1-KO parasites produced significantly fewer oocysts than control parasites (Figure 1C), consistent with the observed reduction in ookinete formation. Despite similar oocyst sizes between mutant and control parasites (Figure 1D), live imaging was consistent with a mild developmental delay in TEX1-KO oocysts, as fewer nuclei were observed at time-matched analysis in TEX1-KO oocysts (Figure 1E). By day 14, mutant oocysts displayed disorganized sporozoite formation and aberrant nuclear morphology (Figure 1F, white arrows).

Sporozoite quantification confirmed the impaired development. TEX1-KO parasites produced fewer midgut (Figure 1G) and therefore less salivary gland sporozoites (Figure 1H) than the control; although surface localization of circumsporozoite protein (CSP) appeared similar as wildtype (Figure 1I).

To further investigate whether the TEX1 deficiency in sexual development and oocyst maturation can be compensated, i.e., rescued by co-infection with wildtype parasites, we employed the “Gene Out Marker Out” (GOMO) strategy [37] (*Pb*GEM-645656). In this approach, TEX1-KO parasites were generated with a fluorescent selection cassette (GFP/hDHFR-yFCU/mCherry), enabling us to track and distinguish them from co-infecting wildtype (*Pb*WT) parasites. Mice were infected with a 1:1 mixture of TEX1-KO GOMO and *Pb*WT blood-stage parasites. After three days of infection, mature gametocytes from both parasite lines were confirmed microscopically, and mosquitoes were then allowed to feed on the animal. If TEX1 function was dispensable in the diploid or polyploid stages, or if wildtype gametes could complement the defect in trans (e.g., by forming heterozygous zygotes), one would expect the presence of wildtype parasites to rescue the TEX1-KO phenotype.

However, oocyst counts by fluorescence in mosquitoes fed on co-infected mice were significantly reduced, and the proportion of TEX1-KO parasites to the resulting sporozoite population remained very low (Supplementary Figure S3A,B). This clearly indicated that wildtype parasites were unable to rescue the developmental defects of the TEX1-KO mutants. These findings suggest that TEX1 function is required already at the haploid sexual (gametocyte/gamete) stage, thus before fertilization, or that even in the diploid zygote, the presence of a single functional TEX1 allele from one parent is insufficient for normal development. Thus, TEX1 appears to act either at an early, pre-fertilization stage in each individual parasite or in a dosage-sensitive, non-redundant manner during zygote and early oocyst development.

### 3.2. TEX1 Is Important for Parasite Survival and Maturation During Liver Stage Development

To evaluate the role of TEX1 during the vertebrate liver stage, the infectivity and development of TEX1-KO sporozoites in vitro and in vivo were tested. HeLa cells were infected with 20,000 salivary gland sporozoites from either TEX1-KO or *Pb*1868 parasites, and exoerythrocytic forms (EEFs) development was analyzed over time.

**Figure 2:**
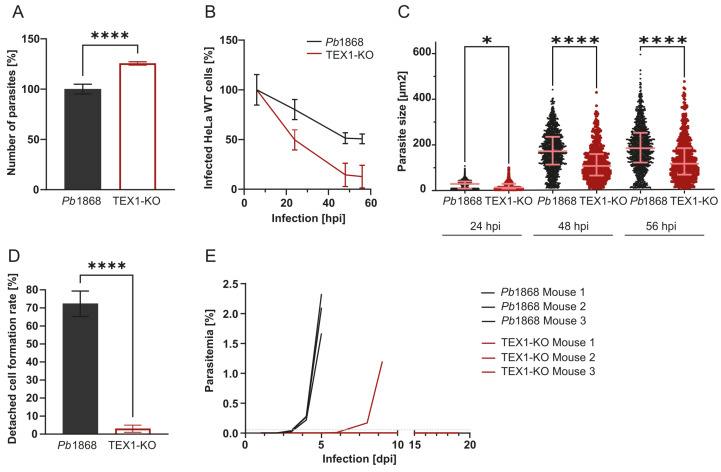
TEX1 is important for parasite survival and development during the liver stage. (**A**) Quantification of intracellular parasites at 6 hpi in HeLa cells infected with *Pb*1868 or TEX1-KO sporozoites. TEX1-KO parasites show a slight increase at this early time point (Student’s *t*-test; **** = *p* < 0.0001). (**B**) Parasite survival over time (6, 24, 48, and 56 hpi), showing a stronger reduction in TEX1-KO parasite numbers compared to *Pb*1868. Data shown are from triplicate wells. (**C**) Size measurements of liver stage parasites at 24, 48, and 56 hpi. TEX1-KO parasites are significantly smaller at later time points (one-way ANOVA; * = *p* < 0.05; **** = *p* < 0.0001; n > 640 parasites). Error bars represent median with interquartile range. (**D**) Detached cell formation at 65 hpi in vitro, expressed as a percentage of parasites present at 48 hpi. TEX1-KO parasites show a dramatic reduction in detached cell formation (Student’s *t*-test; **** = *p* < 0.0001). Data is from three independent replicates. (**E**) Blood stage parasitemia in C57BL/6 mice injected intravenously with 5000 salivary gland sporozoites from *Pb*1868 or TEX1-KO parasites. Measurements are considered positive if parasitemia reaches 0.01% (represented by the dotted line in grey). While all control-infected mice developed parasitemia by day 3, only one out of three TEX1-KO–infected mice became blood stage parasite-positive with a delay of 3 days compared to control mice. Parasitemia was monitored daily by flow cytometry until day 19 post-infection.

At 6 hpi, the number of intracellular TEX1-KO parasites was slightly higher than the control, suggesting that initial hepatocyte invasion was not impaired (Figure 2A). However, temporal quantification revealed a marked survival defect: while ~50.7% of *Pb*1868 parasites persisted until 56 hpi, only ~12.7% of TEX1-KO parasites survived to this timepoint (Figure 2B), indicating a progressive loss of viability during development.

Live quantitative imaging showed that TEX1-KO EEFs were significantly smaller than the control at 24, 48, and 56 hpi indicating arrested or impaired developmental growth (Figure 2C). To determine whether TEX1-KO parasites can complete liver stage development and produce detached cells, we quantified detached cell formation at 65 hpi, a process that occurs after PVM rupture, when the parasite triggers host cell rounding and detachment. While ~72% of control EEFs counted at 48 hpi progressed to detached cell formation, only ~3% of TEX1-KO parasites did so (Figure 2D), indicating a strong, near-complete developmental block.

To test the capacity of TEX1-KO parasites to initiate blood stage infection in vivo, C57BL/6 mice were injected intravenously with 5000 salivary gland sporozoites. Infections were monitored daily by flow cytometry. Mice infected with *Pb*1868 sporozoites developed asexual blood stage infection by day 3 post-injection. In contrast, among the three mice infected with TEX1-KO sporozoites, only one developed a blood stage parasitemia, however, with a delay of 3 days (Figure 2E).

Together, these data demonstrate that TEX1-deficient parasites invade hepatocytes but are severely impaired during liver stage development resulting in attenuated progression to the blood stage.

### 3.3. TEX1 Is Required for Endomitosis and Growth During Liver Stage Development

To better understand the nature of the developmental arrest of TEX1-KO parasites during the liver stage, immunofluorescence assays (IFAs) on infected HeLa cells at 6, 24, 48, and 56 hpi were performed by using anti-UIS4 antiserum to visualize the parasitophorous vacuole membrane (PVM) and DAPI to stain host and parasite nuclei. The parasite morphology was monitored by detecting the cytosolic mCherry.

**Figure 3:**
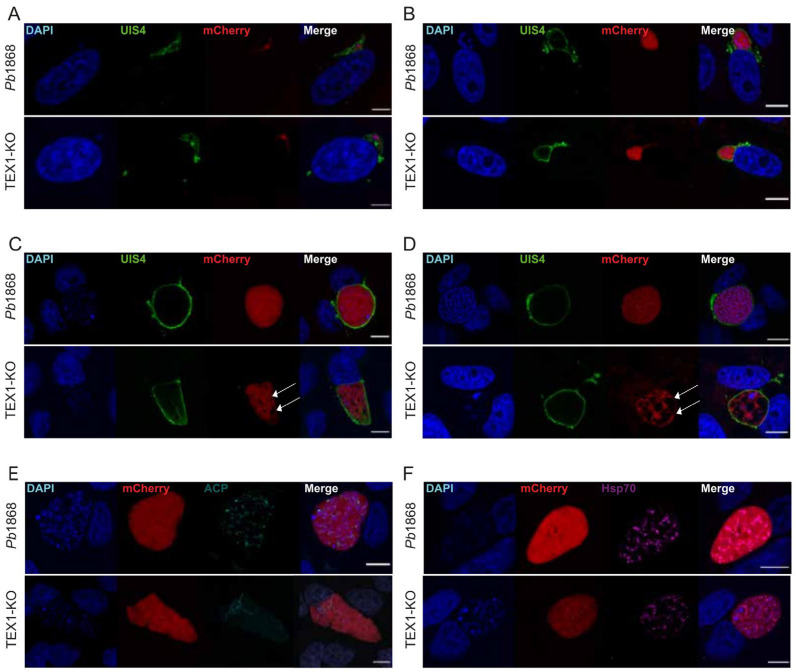
TEX1 is required for parasite nuclear division and growth during *P. berghei* liver stage development. (**A**–**D**) Representative immunofluorescence images of *Pb*1868 and TEX1-KO liver stage parasites at (**A**) 6 hpi (**B**) 24 hpi (**C**) 48 hpi and (**D**) 56 hpi in HeLa cells. Both parasite lines form a PVM, as indicated by UIS4 staining (green). Nuclei are stained with DAPI (blue), and parasite cytoplasm is visualized via mCherry (red). Scale bar = 5 μm (**A**) and 10 μm (**B**–**D**). TEX1-KO parasites show fewer nuclei and abnormal morphology compared to *Pb*1868. Large vacuole-like structures are visible in the cytoplasm of TEX1-KO parasites at late time points, indicative of cell death (white arrows). UIS4 (green), DAPI (blue), mCherry (red), ACP (apicoplast marker) (cyan). Scale bars = 10 μm. (**E**) Apicoplast morphology at 56 hpi. In control parasites, the apicoplast extends throughout the cytoplasm (anti-ACP, cyan), while TEX1-KO parasites show a clumped apicoplast, indicating defective organelle elongation. DAPI (blue), mCherry (red), ACP (cyan). Scale bar = 10 μm. (**F**) Mitochondrial morphology at 56 hpi. Both *Pb*1868 and TEX1-KO parasites show similar mitochondrial structure (anti-TgHSP70, magenta), suggesting that TEX1 is not required for mitochondrial development. DAPI (blue), mCherry (red). Scale bar = 10 μm.

At 6 and 24 hpi, both control and TEX1-KO parasites successfully formed and maintained a PVM (Figure 3A and Figure 3B, respectively), indicating that TEX1 is not required for early invasion or vacuole establishment. However, at 48 and 56 hpi, TEX1-KO parasites exhibited major defects in nuclear division and growth (Figure 3C and Figure 3D, respectively). Mutant parasites displayed fewer nuclei with a majority of them larger and less compact (Figure 3D). In many cases, large vacuole-like dark regions were visible within the mCherry-labelled cytoplasm, suggesting parasite death or degeneration (Figure 3D, white arrow mCherry image).

To assess whether these developmental defects extended to organelle biogenesis, we examined the morphology of the apicoplast (ACP signal) and mitochondrion (*Tg*Hsp70 signal) at 56 hpi. While control parasites showed an extended and reticulated apicoplast structure, the apicoplast in TEX1-KO parasites appeared clumped and failed to elongate throughout the cytoplasm (Figure 3E, selected images highlighting organelle biosynthesis in rare surviving parasites). In contrast, the mitochondrial network appeared unaffected by the TEX1 deletion as attested by a mitochondrial morphology comparable to that of control parasites (Figure 3F, selected images of surviving parasites).

These results indicate that TEX1 is important for proper endomitosis and overall parasite growth during liver stage. Although mitochondria appear unaffected in the few surviving parasites and the PVM remains intact, TEX1 deficiency consistently causes defects in nuclear replication/division and apicoplast extension, ultimately resulting in parasite death at late exoerythrocytic stages.

### 3.4. C-Terminal Tagging of TEX1 Reveals Nuclear Localization

**Figure 4:**
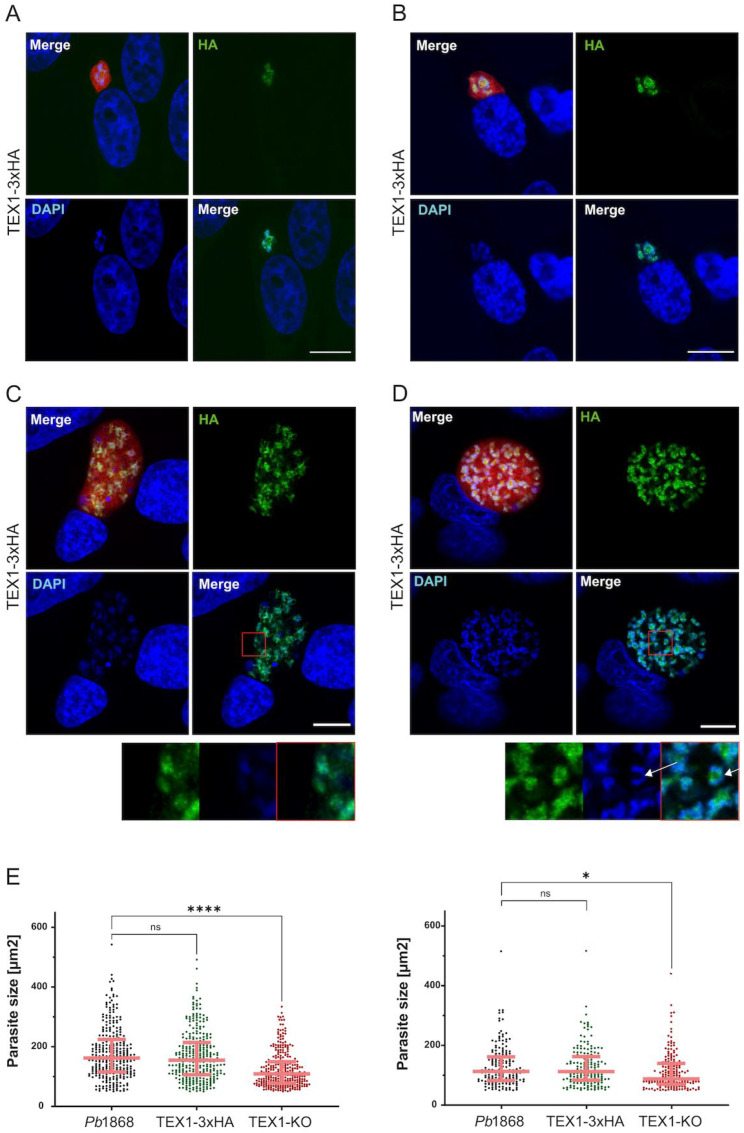
C-terminal 3xHA tagging of TEX1 reveals nuclear localization and allows normal parasite development during the liver stage. (**A**,**B**) Representative immunofluorescence images of TEX1-3xHA–tagged parasites at 24 hpi in HeLa cells. Images are shown with enhanced HA signal for visualization. TEX1-3xHA localizes in discrete subnuclear regions. (**C**,**D**) Representative immunofluorescence images of TEX1-3xHA–tagged parasites at 48 hpi in HeLa cells (**top**) with corresponding zoomed-in views (**bottom**). At this stage, TEX1-3xHA localizes to the nucleus during interphase (**C**) and accumulates both within the nuclei and in interchromosomal regions during metaphase (**D**), as indicated by white arrows. Because nuclear division in *Plasmodium* schizonts is asynchronous, nuclei within the same parasite can be observed in different phases of mitosis. Staining includes DAPI (DNA, blue), anti-HA (3xHA, green), and mCherry (parasite, red). Scale bar = 10 μm. (**E**) Size measurements of liver stage parasites at 48 hpi in HeLa cells (**left**) and in primary hepatocytes (**right**). Statistical analysis by one-way ANOVA; * = *p* < 0.05; **** = *p* < 0.0001; n = 300 parasites for HeLa cells and n > 170 parasites for primary hepatocytes; data pooled from 3 independent replicates). Error bars represent median with interquartile range.

To investigate the subcellular localization of TEX1 during liver stage development, we first generated parasites expressing TEX1 fused to a C-terminal GFP tag under endogenous regulatory control (Supplementary Figure S4). TEX1-GFP fluorescence could be detected early during liver-stage development and, in many parasites, localized prominently to discrete subnuclear foci that strongly resembled the pattern later observed in the TEX1-3×HA line (Figure 4). These parasites proceeded through early rounds of nuclear replication and exhibited overall normal morphology.

However, TEX1-GFP expression also showed marked heterogeneity. A subset of parasites displayed aberrant or diffuse TEX1-GFP staining patterns that did not clearly coincide with nuclei (Supplementary Figure S4). These parasites frequently failed to expand and did not progress to mature schizonts. These observations suggested that the GFP moiety may interfere with TEX1 function or localization in a fraction of parasites.

This heterogeneity complicated the interpretation of TEX1-GFP parasites and prompted us to generate an alternative endogenously tagged line using the smaller 3×HA epitope to determine TEX1 localization with minimal perturbation.

To obtain a reliable and function-preserving readout of TEX1 localization, we generated TEX1-3×HA parasites. In contrast to the heterogeneity observed in the TEX1-GFP line, TEX1-3×HA parasites developed normally, underwent repeated rounds of nuclear division, and produced mature schizonts. Immunostaining revealed a strong and sharply defined TEX1-HA signal exclusively within the parasite nucleus, forming discrete subnuclear foci at all analyzed time points (24–54 hpi) (Figure 4). These foci were observed both during interphase and in the interchromosomal region during mitosis, consistent with TEX1 participating in nuclear processes linked to replication or chromatin organization.

Importantly, the TEX1-HA pattern closely matched the subnuclear foci observed in the subset of TEX1-GFP parasites with normal development, providing consistent evidence that TEX1 resides in specific nuclear domains that likely correspond to functional sites involved in endomitosis.

Together, these data validate TEX1-3×HA as a reliable tool for studying TEX1 localization and indicate that the variability observed in TEX1-GFP parasites reflects tag-specific interference rather than biological heterogeneity.

## 4. Discussion

The ubiquitin system plays a central role in post-translational regulation of proteins [39], impacting processes ranging from proteasomal degradation to transcriptional control and cell cycle progression [40,41,42]. Within this system, E3 ubiquitin ligases such as those of the RING family act as key substrate-specific adaptors. In *Plasmodium*, much of the ubiquitin machinery has been characterized in the asexual blood stages [27,43,44,45], while its functions during pre-erythrocytic development remain largely unexplored. Here, we identify the RING-type E3 ligase TEX1 as a critical regulator of *P. berghei* development during both mosquito and liver stages. TEX1 knockout parasites exhibited pronounced defects in sexual reproduction, with a strong reduction in ookinete formation and oocyst maturation. In the mosquito midgut, TEX1-deficient oocysts showed aberrant nuclear morphologies and reduced sporozoite output, highlighting a role for TEX1 in sporogony. Importantly, a rescue experiment using co-infection with wildtype parasites failed to restore sporozoite numbers, indicating that TEX1 is required in a cell-autonomous manner, likely before or during fertilization, and that a single wildtype allele is insufficient for complementation.

The phenotype in liver stages was even more striking. Although TEX1-KO sporozoites successfully invaded hepatocytes and formed a PV, they exhibited impaired DNA replication, reduced parasite size and markedly decreased survival over time relative to *Pb*1868. Only a small fraction of parasites progressed into mature liver stage forms in vitro, which is in line with the strong delay in forming a measurable blood stage infection. These findings point to TEX1 as a critical regulator of exoerythrocytic schizogony.

To define TEX1 localization and assess its functional requirements, we initially generated a TEX1-GFP parasite line. TEX1-GFP revealed a clear nuclear localization pattern in many parasites, closely resembling the staining seen in the TEX1-3×HA line. However, a substantial subset of TEX1-GFP parasites showed aberrant or mislocalized fluorescence and did not successfully complete liver-stage development. This heterogeneity strongly suggested that the GFP tag interferes with TEX1 function or stability in a fraction of parasites, complicating interpretation of the GFP data.

To overcome these limitations, we generated a TEX1-3×HA line using the much smaller HA epitope. TEX1-HA parasites developed normally through the liver stage and exhibited a consistent and sharply defined nuclear localization pattern. This confirms that TEX1 is a nuclear protein and demonstrates that the HA tag preserves TEX1 function without detectable perturbation. Importantly, the nuclear foci observed in functional TEX1-HA parasites match the localization seen in the subset of TEX1-GFP parasites that developed normally, further validating the overall localization pattern.

The combined GFP and HA data indicate that TEX1 is a nuclear protein whose function is sensitive to the nature of the fusion partner. While GFP tagging introduces artefactual phenotypes in a portion of parasites, HA tagging provides a reliable and physiologically meaningful tool for mechanistic investigation. These findings reinforce TEX1’s role as a regulator of nuclear biology during liver-stage schizogony and underscore the importance of tag choice when characterizing essential nuclear proteins in *Plasmodium*.

The nuclear localization of TEX1, combined with its critical role in parasite replication, suggests parallels with the yeast ortholog Bre1 [46,47], to which TEX1 was assigned based on sequence similarity, although this has not yet been functionally validated.

In yeasts, Bre1 mediates monoubiquitination of histone H2B in coordination with the E2 enzyme Rad6, promoting chromatin remodeling and facilitating transcriptional activation [46,48], DNA replication, and double-strand break formation during meiosis [48,49]. TEX1 may fulfill a similar function in *Plasmodium*, regulating chromatin structure or gene expression programs required for *Plasmodium* unconventional cell cycle. The presence of large vacuolar structures and arrested growth in TEX1-deficient parasites is consistent with replication stress and genome instability described in Bre1-deficient systems [48,50].

Interestingly, our observations that the deletion of TEX1 disturbs nuclear division and apicoplast segregation, while leaving mitochondrial morphology unaffected, align with recent ultrastructural work in *P. falciparum* asexual blood stages showing that centriolar plaques (CPs) physically associate with nuclei and apicoplasts [51,52] but not with mitochondria [52]. In that study, Verhoef et al. demonstrated that apicoplast segregation depends on CPs, whereas mitochondrial fission occurs later and independently of these structures. Association between centrosome and apicoplast has also been observed in *Toxoplasma* [53]. The congruence between these findings suggests that TEX1 may act, directly or indirectly, at the CP to coordinate segregation of nuclei and apicoplasts. Our data therefore raises the possibility that TEX1 contributes to CP-linked mechanisms controlling nuclear and apicoplast inheritance during liver-stage schizogony, while the mitochondrion remains largely unaffected because it segregates through a distinct, CP-independent pathway.

The role of TEX1 described here for *P. berghei* differs from the initial characterization of its *P. falciparum* orthologue, which was reported as a PEXEL-negative exported protein associated with MAHRP1 at Maurer’s clefts in the infected erythrocyte cytosol [30]. Several factors may account for this apparent discrepancy. First, the life-cycle stages examined differ markedly: the earlier work focused on asexual blood stages of *P. falciparum*, whereas our study interrogates mosquito and liver stages of *P. berghei*, where nuclear division and centriolar-plaque-linked segregation of nuclei and apicoplasts dominate [13,15,52,54]. Second, *Plasmodium* species often show stage- and species-specific repurposing of conserved proteins, and divergent transcription profiles of TEX1 orthologues have already been noted across species. Finally, it is possible that TEX1 carries out multiple, context-dependent functions, export-related in the *P. falciparum* blood stage, but required for organellar biogenesis and segregation during *P. berghei* liver schizogony. Thus, rather than being mutually exclusive, the two sets of observations may reflect complementary aspects of TEX1 biology across parasite lineages and developmental stages.

Overall, our data establishes TEX1 as a key regulator of karyokinesis and parasite maturation during liver stage development. Although rare breakthrough blood-stage infections can occur following TEX1 deletion, liver-stage development is profoundly impaired, resulting in a severe and biologically meaningful developmental bottleneck. From a therapeutic perspective, this distinction is important: a factor does not need to be strictly essential for survival to constitute a valuable drug target. Proteins whose inhibition causes a severe developmental bottleneck can effectively halt transmission and prevent progression to symptomatic blood-stage infection. TEX1 satisfies several key criteria for such an intervention point: it is parasite-encoded, shows no obvious functional redundancy, operates during a clinically silent but biologically indispensable stage, and is highly sensitive to structural perturbation, as demonstrated by the dominant-negative phenotype of the tagged protein. Targeting TEX1 or its interactions with the ubiquitin machinery may therefore offer a strategy to selectively block liver stage maturation and interrupt transmission. Future work should focus on identifying TEX1 substrates and evaluating whether selective inhibitors of its E3 activity can prevent liver stage progression without affecting host ubiquitin pathways.

## Data Availability

All data supporting the findings of this study are included within the manuscript. No additional datasets were generated or analyzed during the current study.

## Supplementary Materials

The following supporting information can be downloaded at: https://www.mdpi.com/article/10.3390/cells15020155/s1, Figure S1. InterPro-annotated RING-type domain for TEX1. (**A**) TEX1 domain predictions were annotated using external signature accession numbers (primary IDs), with corresponding annotation identifiers (secondary IDs), functional descriptions, and the start and end positions within the amino acid sequence, along with their expected values (E-values) (data adapted from PlasmoDB [47]). (**B**) Schematic representation of the domains presented in (**A**). (**C**) AlphaFold structural prediction (left) and Predicted Aligned Error (PAE) plot (right) of TEX1 amino acid sequence [55]. (**D**) AlphaFold structural prediction (left) and Predicted Aligned Error (PAE) plot (right) of TEX1-3xHA (D) amino acid sequence [55]. Figure S2. Generation and confirmation of TEX1 knockout parasite lines. (**A**) Schematic representation (left) of the strategy used to generate the TEX1-KO parasite line by double homologous recombination. The KO cassette (PbGEM-266212) replaces the endogenous PBANKA_0102200 gene with a 3xHA-hDHFR-yFCU selection marker and a gene-specific barcode. Confirmation of successful integration (right) was performed by diagnostic PCR. Grey arrow: PBANKA_0102200 coding sequence; red bar: barcode; blue arrows: primer binding sites. (**B**) Schematic representation (left) of the "Gene Out Marker Out" (GOMO) strategy to generate a TEX1-KO GOMO parasite line using the PbGEM-645656 vector. The endogenous PBANKA_0102200 locus is replaced by a triple marker cassette (GFP/hDHFR-yFCU/mCherry). Confirmation of integration (right) was performed by PCR. Grey arrow: PBANKA_0102200; blue arrows: primer binding sites. (**C**) Generation (left) and confirmation (right) of a TEX1-3x HA parasite line by double homologous recombination. The endogenous PBANKA_0102200 gene is followed by hDHFR-yFCU and mCherry selection marker after successful integration. Grey arrow: PBANKA_0102200 gene; blue arrow: primer binding site, UTR = untranslated region; DHFR = dihydrofolate reductase; hsp70 = heat shock protein 70 kDa; AMP = ampicillin. Figure S3: Rescue of TEX1-KO GOMO with PbWT parasites in the diploid stage of the Plasmodium parasite did not result in increased oocyst numbers in the mosquito midgut. (**A**) Quantification of midgut oocysts from mosquitoes infected with Pb1868, TEX1-KO GOMO alone, or TEX1-KO GOMO co-infected with wildtype (PbWT) parasites, on days 6 and 14 post-feed. Oocyst numbers remained significantly reduced in both TEX1-KO conditions, with or without PbWT (one-way ANOVA; *** = *p* < 0.001; **** = *p* < 0.0001; ns = not significant; n = 10 midguts per group). (**B**) Quantification of salivary gland sporozoites per mosquito at days 17–20 for Pb1868, TEX1-KO GOMO alone, and co-infection with PbWT. Total sporozoite numbers were significantly reduced in the TEX1-KO conditions (one-way ANOVA; *** = *p* < 0.001; ns = not significant; n = 10–30 mosquitoes per group). (**C**) Proportional contribution of TEX1-KO GOMO and PbWT sporozoites in co-infected mosquitoes, showing that TEX1-KO parasites make up only a small fraction of the sporozoite population. Figure S4. TEX1-EGFP localizes in liver stage development primarily near the nucleus. (**A**) Schematic representation of the strategy used to generate the TEX1-EGFP parasite line by single homologous recombination. The endogenous PBANKA_0102200 gene is followed by an EGFP and DHFR-yFCU selection marker after successful integration. Grey arrow: PBANKA_0102200 coding sequence; blue arrows: primer binding sites; AMP = ampicillin. (**B**) Confirmation of successful integration was performed by diagnostic PCR. (**C**) Representative immunofluorescence images of TEX1-EGFP liver stage parasites at 30 (top), 48 hpi (middle) and 56 hpi (bottom) in HeLa cells. TEX1-EGFP localizes primarily near the nucleus. Nuclei are stained with DAPI (blue), TEX1-EGFP is visualized via GFP (green) and parasite cytoplasm via mCherry (red) and the Endoplasmatic reticulum (BiP, yellow). Scale bar = 10 μm.
