# Supplementary material for: The Putative E3 Ubiquitin Ligase TEX1 Is Required for Nuclear Biology and Developmental Progression of Plasmodium berghei in the Liver
Source: Cells. 2026 Jan 15;15(2):155. doi: 10.3390/cells15020155 (PMC12839800; doi:10.3390/cells15020155)
Supplement: Supplementary file 1 [file cells-15-00155-s001.zip › cells-3969858-supplementary.pdf]

A

Primary ID	Secondary ID	Description	Start	End	E-value
PTHR23163	RING FINGER PROTEIN-RELATED	E3 ubiquitin ligase Bre1	31	899	7.2E-24
PTHR23163:SF0	E3 UBIQUITIN-PROTEIN LIGASE BRE1	E3 ubiquitin ligase Bre1	31	899	7.2E-24
PF13920	ZINC FINGER C3HC4	N/A	846	891	1.4E-09
SM00184	RING	Zinc finger, RING-type	848	886	9.1E-08
SSF57850	RING/U-box	N/A	841	898	2.4E-15

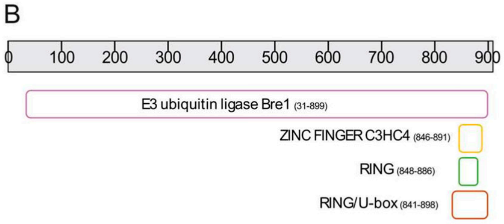

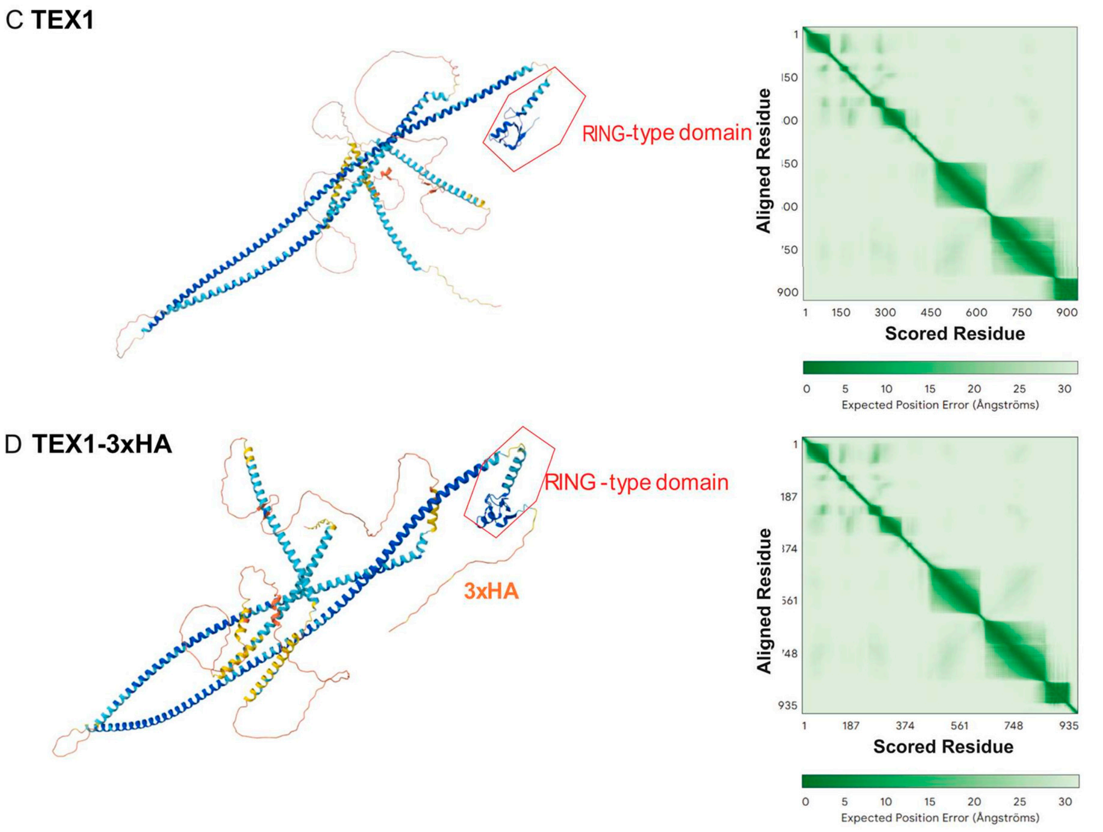

Supplementary Figure S1. AlphaFold structure and prediction of TEX1 with InterPro-annotated RING-type domain. (**A**) TEX1 domain predictions were annotated using external signature accession numbers (primary IDs), with corresponding annotation identifiers (secondary IDs), functional descriptions, and the start and end positions within the amino acid sequence, along with their expected values (E-values) (data adapted from PlasmoDB [47]). (**B**) Schematic representation of the domains presented in (A). (**C**) AlphaFold structural prediction (left) and Predicted Aligned Error (PAE) plot (right) of TEX1 amino acid sequence [55]. (**D**) AlphaFold structural prediction (left) and Predicted Aligned Error (PAE) plot (right) of TEX1-3xHA (D) amino acid sequence [55].

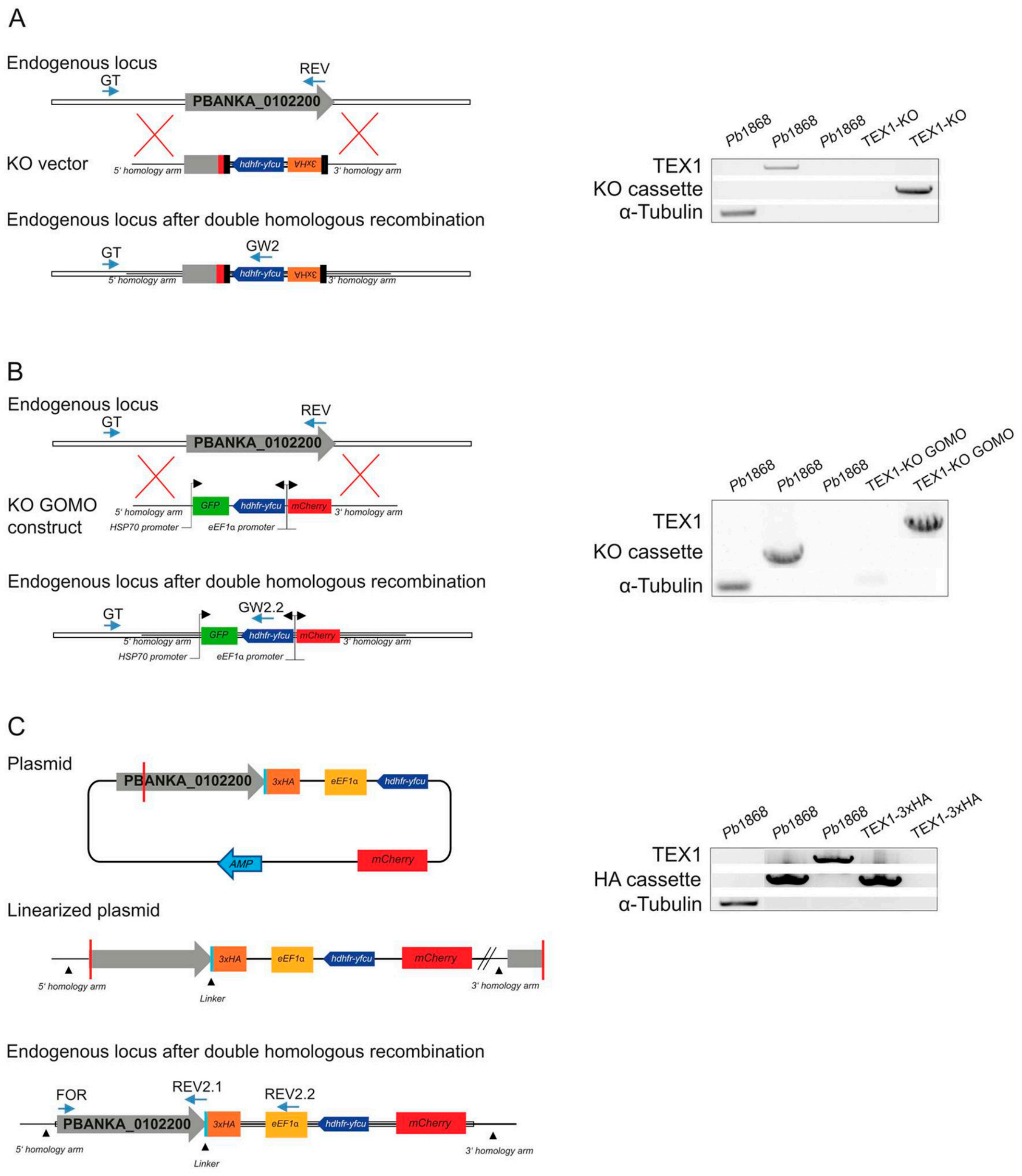

Supplementary Figure S2: Generation and confirmation of TEX1 knockout parasite lines.
(**A**) Schematic representation (left) of the strategy used to generate the TEX1-KO parasite line by double homologous recombination. The KO cassette (PbGEM-266212) replaces the endogenous PBANKA_0102200 gene with a 3xHA-hDHFR-yFCU selection marker and a gene-specific barcode. Confirmation of successful integration (right) was performed by diagnostic PCR. Grey arrow: PBANKA_0102200 coding sequence; red bar: barcode; blue arrows: primer binding sites. (**B**) Schematic representation (left) of the "Gene Out Marker Out" (GOMO) strategy to generate a TEX1-KO GOMO parasite line using the PbGEM-645656 vector. The endogenous PBANKA_0102200 locus is replaced by a triple marker cassette (GFP/hDHFR-yFCU/mCherry). Confirmation of integration (right) was performed by PCR. Grey arrow: PBANKA_0102200; blue arrows: primer binding sites. (**C**) Generation (left) and confirmation (right) of a TEX1-3x HA parasite line by double homologous recombination (grey arrow = PBANKA_0102200 gene; blue arrow = primer binding site).

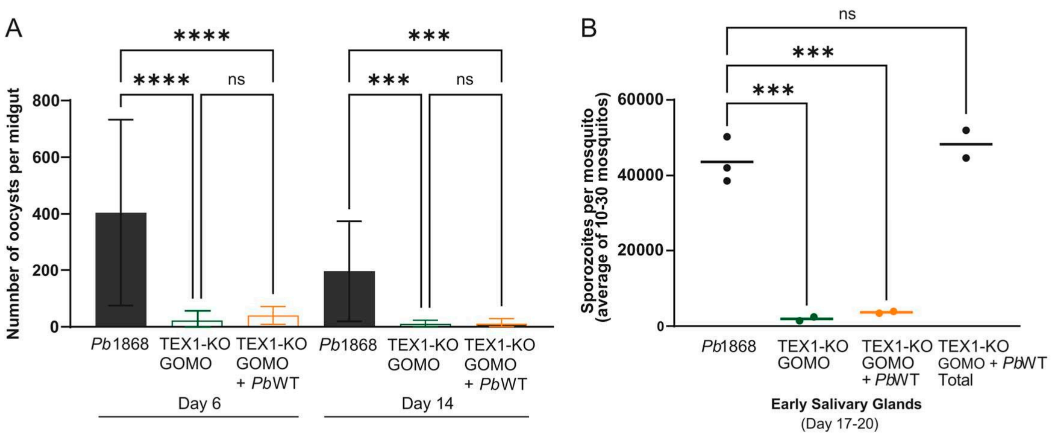

Supplementary Figure S3: Rescue of TEX1-KO GOMO with *Pb*WT parasites in the diploid stage of the *Plasmodium* parasite did not result in increased oocyst numbers in the mosquito midgut. (**A**) Quantification of midgut oocysts from mosquitoes infected with *Pb*1868, TEX1-KO GOMO alone, or TEX1-KO GOMO co-infected with wildtype (*Pb*WT) parasites, on days 6 and 14 post-feed. Oocyst numbers remained significantly reduced in both TEX1-KO conditions, with or without *Pb*WT (one-way ANOVA; *** = $P < 0.001$; **** = $P < 0.0001$; n= 10 midguts per group). (**B**) Quantification of salivary gland sporozoites per mosquito at days 17– 20 for *Pb*1868, TEX1-KO GOMO alone, and co-infection with *Pb*WT. Total sporozoite numbers were significantly reduced in the TEX1-KO conditions (one-way ANOVA; *** = $P < 0.001$; ns = not significant; n = 10–30 mosquitoes per group).

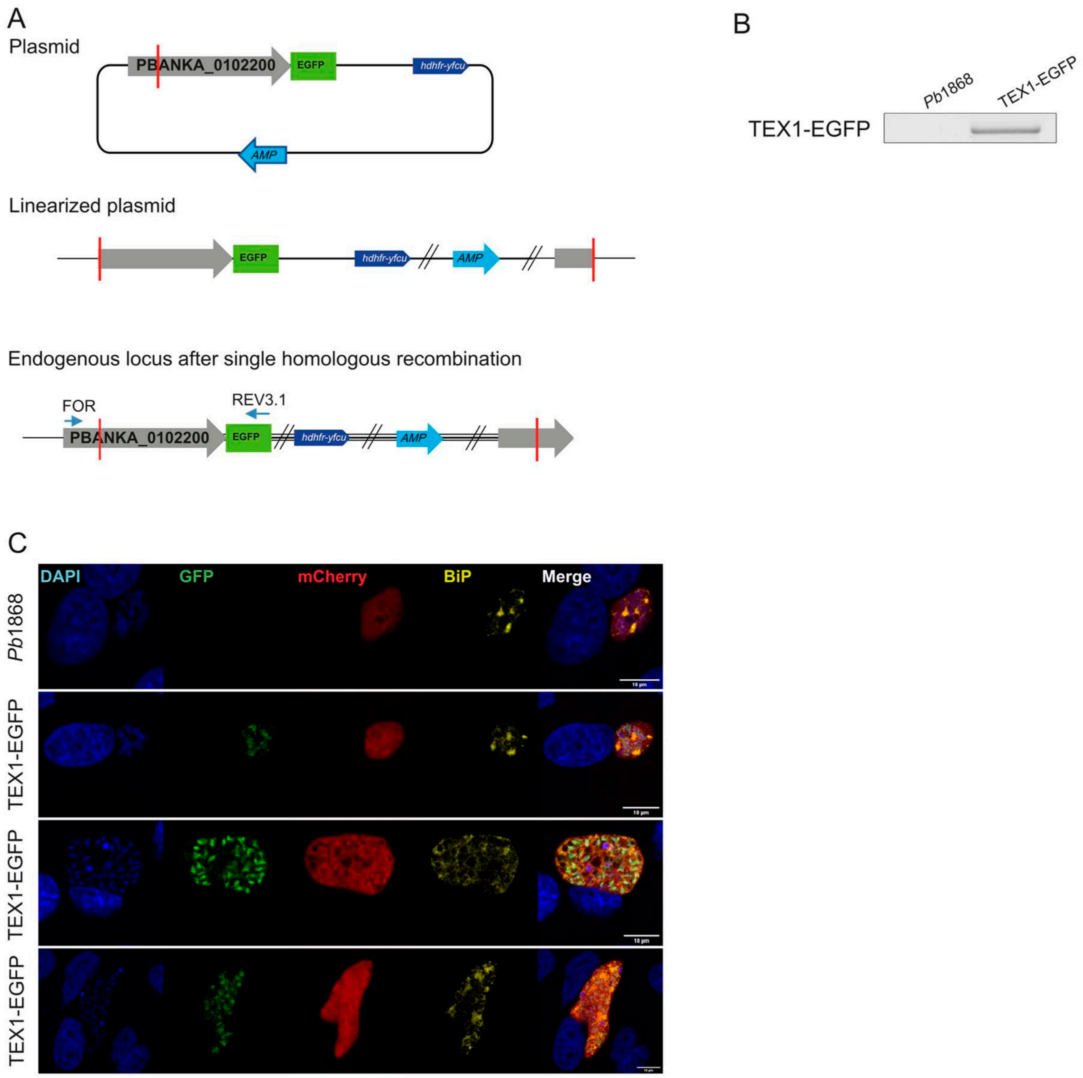

Supplementary Figure S4: TEX1-EGFP localizes in liver stage development primarily near the nucleus. (**A**) Schematic representation of the strategy used to generate the TEX1-EGFP parasite line by single homologous recombination. The endogenous PBANKA_0102200 gene is followed by an EGFP and DHFR-yFCU selection marker after successful integration. Grey arrow: PBANKA_0102200 coding sequence; blue arrows: primer binding sites. (**B**) Confirmation of successful integration was performed by diagnostic PCR. (**C**) Representative immunofluorescence images of TEX1-EGFP liver stage parasites at 30 (top), 48 hpi (middle) and 56 hpi (bottom) in HeLa cells. TEX1-EGFP localizes primarily near the nucleus. Nuclei are stained with DAPI (blue), TEX1-EGFP is visualized via GFP (green) and parasite cytoplasm via mCherry (red) and the Endoplasmatic reticulum (BiP, yellow). Scale bar = 10 µm.
